# Laparoscopic-Assisted Single-Port Appendectomy in Children: It Is a Safe and Cost-Effective Alternative to Conventional Laparoscopic Techniques?

**DOI:** 10.1155/2013/165108

**Published:** 2013-12-08

**Authors:** Sergio B. Sesia, Frank-Martin Haecker

**Affiliations:** Department of Pediatric Surgery, University Children's Hospital Basel, Spitalstrasse 33, 4056 Basel, Switzerland

## Abstract

*Aim*. Laparoscopic-assisted single-port appendectomy (SPA), although combining the advantages of open and conventional laparoscopic surgery, is still not widely used in childhood. The aim of this study was to evaluate the safety and the cost effectiveness of SPA in children. *Methods*. After institutional review board approval, we retrospectively evaluated 262 children who underwent SPA. The appendix was dissected outside the abdominal cavity as in open surgery. For stump closure, we used two 3/0 vicryl RB-1 sutures. *Results*. We identified 146 boys (55.7%) and 116 girls (44.3%). Median age at operation was 11.4 years (range, 1.1–15.9). Closure of the appendiceal stump using two sutures (cost: USD 15) was successful in all patients. Neither a stapler (cost: USD 276) nor endoloops (cost: USD 89) were used. During a follow-up of up to 69 months (range, 30–69), six obese children (2.3%, body mass index >95th percentile) developed an intra-abdominal abscess after perforated appendicitis. No insufficiency of the appendiceal stump was observed by ultrasound. Five of them were treated successfully by antibiotics, one child required drainage. *Conclusion*. The SPA technique with conventional extracorporal closure of the appendiceal stump is safe and cost effective. In our unit, SPA is the standard procedure for appendectomy in children.

## 1. Introduction

Since the first description of laparoscopic appendectomy by Semm in 1983 [1], several laparoscopic techniques have evolved to attain stump closure. Surgeons can choose between clip, stapler, endoloops [2, 3], or simple sutures as in open surgery. Commonly, endoloops or endostaplers are used for closing the stump of the appendix [3, 4]. We report about the cost of appendiceal stump closure using only sutures in laparoscopic-assisted single-port appendectomy (SPA) in children.

## 2. Materials and Methods

After institutional review board approval, we retrospectively reviewed the medical records of children who underwent SPA between August 2005 and December 2008 at the University Children's Hospital of Basle (UKBB). According to the World Organization of Gastroenterology Research Committee [5], diagnosis of acute appendicitis was made by comprehensive anamnesis, physical examination with particular attention of rebound tenderness on the right lower abdominal quadrant, supporting laboratory tests such as white blood cell count (WBC) and C-reactive protein (CRP), and ultrasound scan of the abdomen. All children admitted on our emergency room with suspected acute appendicitis were considered for SPA and included in this study. The only exclusion criterion was appendectomy performed by an open surgical approach. Surgery was performed under the supervision of five board-certified pediatric surgeons. Extracorporal sutures closed the stumps of all appendices, including perforated appendicitis. Neither endoloops nor endostaples were used. The SPA with extracorporal stump closure represented the standard technique for appendectomy. The main purpose of this retrospective single-center study was to analyze the cost for closing the appendiceal stump. Other material and personnel costs for surgery, anesthesia, and costs for operating room and for hospital stay were not considered. Data were stored in an Excel database (Microsoft Corporation, Redmond, WA).

## 3. Surgical Technique

As previously described [6], SPA was performed using one 12-mm single-use balloon-trocar (Auto Suture, United States Surgical/Tyco Healthcare, Type OMS-T10BT, Norwalk, CT) with one conventional laparoscopic forceps (COMEG, Endoskopie GmbH & Co., Type PAJUNK 12929410). After introducing the trocar through a subumbilical incision, the appendix was grasped and exteriorized through the umbilicus. Dissection and appendectomy were performed in the standard open fashion. After ligature of the basis of the appendix, one purse-string suture and one z-shaped absorbable suture 3/0 vicryl RB-1 placed through the seromuscular base of the caecum closed the appendiceal stump.

All operations were accomplished on emergency basis. All children with suspected appendicitis were managed according to a standard preoperative protocol such as mechanical cleaning of the umbilicus with noncolored octenidine dihydrochloride (Octenisept) and a loading i.v.-dose of metronidazole and of cefuroxime within 15 minutes before starting surgery [6].

## 4. Results

Between August 2005 and December 2008, 262 children underwent SPA, including 146 males (55.7%) and 116 females (44.3%). Median age at operation was 11.4 years (range, 1.1–15.9). Closure of the appendiceal stump using two vicryl RB-1 sutures at a cost of USD 7.5 each was successful in all patients. Conversion to open appendectomy occurred in 35 children (13.4%) and to conventional 3-trocar laparoscopic appendectomy in 9 children (3.4%). In a previous study, we reported about complications and main outcomes in correlation to histological results [6]. No insufficiency of the appendiceal stump was observed by ultrasound. During a followup of 69 months (range, 30–69), six obese children (2.3%, body mass index > 95th percentile) developed an intraabdominal abscess after perforated appendicitis. One child (0.4%) required surgical drainage, and the other five children (1.1%) responded to conservative treatment. No recurrence of intraabdominal abscess was noted to date. Neither a stapler (cost: USD 276) nor endoloops (cost: USD 89) were used. There was no mortality related to SPA in this series. Median operating time was 55 minutes (range, 15.0–160.0). The median length of hospital stay was 4 days (range, 3.0–18.0). As referred earlier [6], the operating surgeon was in 71.7% a resident under the direct supervision of a board certified senior pediatric surgeon.

## 5. Discussion

The increasing pressure of national healthcare insurance to contain costs of inpatient hospitalization aroused our interest in performing this cost-benefit analysis of SPA. Since this year, diagnosis-related group (DRG) was introduced in Switzerland. Now, a flat rate reimbursement replaced the traditional cost-based reimbursement system called TARMED (Tarif médical) [7, 8].

Appendicitis is the most common cause of acute abdominal disease in children [9]. Despite several advantages of laparoscopic appendectomy (LA) such as less pain, earlier discharge, better cosmesis, and earlier return to normal activities [10], open appendectomy (OA) still represents a standard surgical technique [11, 12]. In particular, SPA has not yet evolved as gold standard for the treatment of acute appendicitis. Compared to OA, LA using the three-trocar technique has been shown to induce less postoperative pain and faster recovery of the bowel function but seems to be associated with a higher rate of intraabdominal abscess formation, especially in perforated appendicitis [13], and with higher costs [14].

The different manner of closing the appendiceal stump may play a role in developing an intraabdominal abscess [13] and influence substantially the cost of LA. The technique of closure of the appendiceal stump in LA varies greatly. Usually, a noninversion of the appendiceal stump is performed in conventional three-trocar LA. This circumstance could explain a higher rate of intraabdominal abscess in conventional LA. Since the introduction of SPA in mid-2005 at our department, the appendiceal stump is ligated, inverted, and closed by one z-shaped suture. As reported earlier [6], we encountered 6 cases of intraabdominal abscess after SPA. All of them occurred in obese children (BMI > 95th percentile) with perforated appendicitis. In four of them, the surgeon carried out a lavage of the peritoneal cavity with saline. Despite a controversial discussion in the literature [15, 16], we hypothesize that the saline lavage may be responsible for bacterial spread throughout the abdomen and the cause of intraabdominal abscess. Due to this experience, we only perform suction of the abdominal fluid collections and no more lavage. A review of the literature shows no significant difference in the incidence of intraabdominal abscess when comparing the suture technique with endoloop and stapler to endoloop only for appendiceal stump closure [17]. But there is a noteworthy difference with regards to the cost.

The decision as to which LA-technique to use depends on its safety and cost. In our opinion, SPA joins the safety of OA (i.e., dissection under direct view) and the advantages of conventional LA (i.e., small skin incision and visibility of the entire abdominal cavity). Different ways to close the appendiceal stump exist such as stapler, clips, endoloop, or endobag [18]. In contrast to several reports of single-port or single-incision laparoscopic appendectomy [19], techniques that involve special trocar, and multiple instruments [20], our SPA-technique requires only one trocar (USD 172) and one conventional laparoscopic instrument and does not necessitate the use of expensive equipment such as retrieval pouch. Regarding these facts, our SPA-technique is less expensive than conventional three-trocar LA reported elsewhere [21, 22]. Closing the appendiceal stump using two 3/0 vicryl RB-1 sutures (USD 15) is 5.9 times less costly than by endoloop and 18.4 times less costly than by stapler.

Our median operating time of 55 min. was slightly higher than those reported in the literature [22], which is related to our learning curve. Especially in complicated appendicitis, the operative time was higher than 55 min., as reported in the literature [23]. Safety of surgical techniques is one of the primary concerns in the literature; the safety of a surgical technique is characterized by its rate of complications.  Table 1 displays the main outcomes of a review of the literature concerning laparoscopic-assisted single-port appendectomy.

The low rate of perioperative complications and of conversions to OA by extension of the subumbilical incision or to conventional LA by the introduction of 2 or more trocars, corroborate the finding that SPA remains a safe operative technique. The safety of OA is commonly accepted, and there are numerous studies underlining the reliability and the safety of LA also in complicated appendicitis in children [15]. However, SPA combines the advantages of both open and laparoscopic surgery and allows for use of both skills in open surgical and laparoscopy techniques. The need for only one single umbilical incision, one conventional laparoscopic instrument without any highly technical devices such as stapler, endoloop, and endobag reduce the time and the mean cost of the SPA-operation. Furthermore, the SPA-technique is extensible allowing additional trocars or devices such as stapler. Notably, SPA can be converted to conventional LA at any time for the treatment of additional pathologies.

## 6. Conclusion

SPA represents an expeditious and reliable technique for appendicitis in pediatric populations. In our opinion, SPA is a safe and cost-effective technique. The main negative features of conventional LA, that are longer operative time and operating room cost compared to OA [24], seem to be not attributable to SPA. Additional randomized trials are needed to verify this hypothesis. In our unit, SPA is the standard procedure for appendectomy in children.

## Figures and Tables

**Table 1:** Recents reports of transumbilical laparoscopic-assisted single-port appendectomy.

Author, year	*n*	Journal	Intraoperative complications	Postoperative complications	Conversions to	
					OA	LA
D'Alessio et al., 2002 [25]	150	Eur J Pediatr Surg	5 (bleeding, rupture appendix)	5 (2 WI, 2 IAA, and 1 omphalitis)	6	6
Pappalepore et al., 2002 [26]	58	Eur J Pediatr Surg	0	0	1	1
Meyer et al., 2004 [27]	163	Zentralbl Chir	0	4 WI, 3 IAA	3	6
Koontz et al., 2006 [22]	111	J Pediatr Surg	0	8 (7 WI, 1 IAA)	2	2
Visnjic 2008 [9]	29	Surg Endosc	n.s.	4 WI	0	0
Sesia et al., 2010 [6]	262	J Laparoendosc Adv Surg Tech	1 serosa lesion	7 (1 WI, 6 IAA)	35	9
Guanà et al., 2010 [28]	231	Afr J Paediatr Surg	n.s.	4 (2 WI)	2	2
Stanfill et al., 2010 [29]	48	J Laparoendosc Adv Surg Tech	0	5 (1 ileus, 1 WI, and 3 IAA)	0	0
Lee et al., 2011 [24]	152	Surg Endosc	0	7 (7 IAA)	0	0
Cobellis et al., 2007 [30]	182	J Laparoendosc Adv Surg Tech	0	2 WI	31	0
Kagawa et al., 2012 [31]	158	Int J Colorectal Dis	0	8 (1 WI, 4 IAA, and 3 ileus)	7	26
Ohno et al., 2012 [21]	416	Surg Endosc	21 (2 serosa lesions, 16 tears of appendix, and 3 bleeding)	77 (31 WI, 21 intestinal obstruction, 15 IAA, 8 enterocolitis, 1 leakage, and 1 stitch abscess)	70	14
Shekherdimian and DeUgarte 2011 [32]	18	Am Surg	n.s.	0	0	0

IAA: intraabdominal abscess, WI: wound infection, n.s.: not specified, OA: open appendectomy, LA: laparoscopic appendectomy.
